# The effects of trauma and non-trauma literature on relieving PTSD of Chinese undergraduates—a randomized controlled trial

**DOI:** 10.3389/fpsyg.2025.1625041

**Published:** 2025-11-28

**Authors:** Ruikai Yuan, Mengjie Li

**Affiliations:** 1 Centre for Research in Language and Linguistics, National University of Malaysia, Bangi, Malaysia; 2 Sichuan University of Media and Communication, Chengdu, China

**Keywords:** PTSD, bibliotherapy, trauma literature, reading self-efficacy, empathy, university students, randomized controlled trial

## Abstract

Traditional psychological interventions for post-traumatic stress disorder (PTSD) face limitations in university settings due to resource constraints, stigma, and low student engagement. This randomized controlled trial explored the effectiveness of reading-based interventions—trauma literature, non-trauma literature, and a control condition—on PTSD symptoms among 105 Chinese university students. Over an 8-week period, the trauma literature group showed the largest reduction in PTSD symptoms (*p* < 0.001), followed by the non-trauma literature group (*p* < 0.001), with minimal change in the control group (*p* < 0.01). Path analysis revealed that reading self-efficacy significantly predicted emotional empathy, which in turn predicted lower PTSD symptoms, indicating a partial mediating effect. These results suggest that exposure to traumatic reading could reduce PTSD symptoms, and structured reading, especially trauma narratives, can serve as a low-cost, scalable intervention to reduce PTSD symptoms and improve emotion regulation through enhanced reading efficacy and empathy. This study offers new evidence supporting bibliotherapy as a feasible and effective alternative for psychological support in higher education.

## 1 Introduction

In recent years, post-traumatic stress disorder (PTSD) has become a major mental health problem for college students, with studies showing that the prevalence of PTSD among Chinese college students ranges from 12 to 39.5% (Xiao et al., 2025; Ghazali et al., 2024). Such discrepancy can be explained by several reasons. For example, surveys conducted in regions heavily affected by public health crises, such as COVID-19 lockdown zones or areas struck by natural disasters, tend to report higher PTSD rates (Wang et al., 2023). Other potential contributors include gender differences (Gao et al., 2020), academic stress (Zhao et al., 2024), family background (Zhang et al., 2021), and varying access to psychological resources across urban and rural background (Liang et al., 2019). Despite the alarming prevalence, the implementation of traditional psychological interventions in university settings still faces great challenges, including limited mental health resources (Ning et al., 2022; Xiang et al., 2018; Shi et al., 2020; Ning et al., 2022) and the stigma of seeking psychological help (Wong et al., 2024; Yu et al., 2022; He et al., 2021). In addition, college students’ busy academic schedules often conflict with treatment requirements (Lei et al., 2021), further reducing participation in mental health interventions. These practical barriers make it necessary to explore some alternative, low-cost, and convenient interventions that can be easily implemented in university settings.

Bibliotherapy—the use of reading materials for therapeutic purposes—has been considered a promising alternative intervention for a number of reasons (Peterkin and Grewal, 2018; Cook et al., 2006). Compared to traditional treatment approaches, reading interventions are low-cost (Sampaio et al., 2016) and require little professional supervision. They also offer greater accessibility and flexibility (James and Römhild, 2024; Veillette et al., 2019), allowing students to engage with treatment content on their own schedule and in a private setting, potentially circumventing barriers associated with stigma.

Bibliotherapy has gained significant attention as a therapeutic approach that utilizes literature to facilitate recovery in mental health.

Research on bibliotherapy has demonstrated its potential efficacy for a variety of psychological disorders, but limited research has specifically explored its effects on post-traumatic stress disorder (PTSD) symptoms in college settings. Furthermore, the psychological mechanisms through which reading interventions may improve trauma symptoms remain poorly understood, hampering efforts to optimize such interventions. Reading self-efficacy, defined as an individual’s belief in their ability to comprehend and engage with literary material, has been linked to greater engagement and deeper emotional involvement with texts (Unrau et al., 2018; Koşar et al., 2022; Aro et al., 2018). High reading self-efficacy may facilitate empathic engagement with characters and narratives (Salama, 2024).

Research has shown that bibliotherapy is clinically effective in treating mild to moderate depression and anxiety symptoms (Khademhoseini et al., 2024; Kramer, 2009; Gregory et al., 2004; Jwaied, 2024; Mazza, 2016; Mazza, 2017; Furman et al., 2002; Sjollema et al., 2012; Van Emmerik et al., 2013). This is further strengthened through the regulation of empathy. Empathy, especially emotional empathy or empathic drive, is a known protective factor in trauma recovery (Wharne, 2022; Wilde, 2019; Liu et al., 2024). Literature can evoke empathy by allowing readers to inhabit another person’s emotional world, a process thought to promote perspective-taking and emotional regulation (LaRusso et al., 2016; Nölke, 2016; Yurdakul et al., 2025; Albrecht, 2016). Several studies suggest that narrative exposure may lead to measurable reductions in PTSD symptoms through such emotional engagement (Glavin and Montgomery, 2017; Jwaied, 2024).

However, few studies have examined its effects on PTSD symptoms or its psychological mechanisms in non-clinical young adult samples. Most existing research focuses on Western contexts, leaving cultural and educational variations largely unaddressed. Furthermore, although empathy and reading self-efficacy have been independently linked to emotional engagement and psychological adjustment, their combined role in mediating the effects of reading interventions on trauma recovery remains unclear. This study aims to evaluate the effectiveness of reading trauma-related vs. non-trauma literature in reducing PTSD symptoms among university students, and to examine the mediating role of empathy in the relationship between reading self-efficacy and PTSD outcomes. The hypotheses of the study are as follow:

*H1*: Trauma literature will produce greater PTSD symptom reduction than non-trauma literature and no-intervention conditions.

*H2*: Reading self-efficacy will be positively associated with emotional empathy.

*H3*: Emotional empathy will be negatively associated with PTSD symptoms.

*H4*: Emotional empathy will mediate the relationship between reading self-efficacy and PTSD symptoms.

## 2 Method

### 2.1 Research design

This study employed a randomized controlled trial design to investigate the effects of different reading interventions on PTSD symptoms among students. Participants were randomly assigned to one of three groups: the trauma literature group (Group 1), the non-trauma literature group (Group 2), and one group without intervention (Group 3). To ensure conceptual clarity, the reading materials were classified into trauma literature and non-trauma literature according to both thematic content and emotional intensity.

Trauma literature in this study refers to narrative texts that explicitly depict personal or collective traumatic experiences such as war, persecution, loss, and survival, and that invite readers to engage in emotional processing through empathy and identification with the narrator’s suffering (Caruth and Hartman, 1996; Felman and Laub, 1992). *The Diary of Anne Frank* was selected as the representative trauma text because it portrays the lived experience of a young girl hiding during the Holocaust, addressing themes of fear, confinement, and resilience in the face of existential threat. In contrast, non-trauma literature refers to narratives that do not directly center on traumatic events but still engage readers in reflective or emotional experiences through universal human themes such as love, imagination, or moral growth. *The Little Prince* was chosen as the non-trauma text because it conveys philosophical and emotional reflection without depicting direct traumatic exposure. Its content elicits gentle emotional resonance rather than distress or re-experiencing, making it suitable as an emotionally engaging yet non-traumatic control narrative.

The study included two measurement time points: baseline (T0) and post-intervention (T1) after 8 weeks, following previous bibliotherapy protocols that suggest 2 months as an optimal duration to observe psychological change while maintaining participant adherence (Arslan et al., 2022a). The group interventions are presented in Table 1.

**Table 1:** Group intervention.

Groups	Reading material	Reading schedule
Group 1	*The Diary of Anne Frank*	1–2 h per week over 8 weeks
Group 2	*The Little Prince*	1–2 h per week over 8 weeks
Group 3	None	

### 2.2 Participants

Participants were recruited from university students aged 18–25. An initial screening was conducted by distributing 1,000 questionnaires. According to existing research, the prevalence of PTSD among Chinese university students is approximately 7–53.8% (Si et al., 2021). From the returned questionnaires, we selected those with scores between 31 and 33 on the PTSD Checklist for DSM-5 (PCL-5), as this range indicates the presence of PTSD symptoms without reaching the threshold that would necessitate clinical intervention (Forkus et al., 2023). Scores above this range were advised to seek professional medical support (Bovin and Marx, 2023). A total of 273 questionnaires met the inclusion criteria. Participants with other severe mental health conditions or those currently undergoing psychotherapy or taking psychotropic medications were excluded. We contacted those who expressed willingness to participate in the experiment. Ultimately, a total of 105 participants were enrolled, with 35 assigned to each group. Randomization was performed using a computer-generated sequence. For participants who exhibited significant emotional responses during the experiment, psychological support was provided to help alleviate distress and ensure their well-being.

### 2.3 Measurements

#### 2.3.1 Reading self-efficacy scale

To measure individuals’ confidence in their ability to understand and appreciate novels, the study adopted a reading self-efficacy scale. Reading self-efficacy was assessed via a four-item scale specifically developed for this study, based on two general self-efficacy measures (Engeser, 2005; Beierlein et al., 2012). The internal consistency of this scale was acceptable (McDonald’s ω = 0.62). Reading self-efficacy was assessed using single items on five-point Likert scales [ranging from “very strongly disagree” (=1) to “very strongly agree” (=5)] (Thissen et al., 2018). The Cronbach’s α of this scale is 0.77, showing an acceptable reliability.

#### 2.3.2 Emotional empathic drive short scale

Empathy was assessed using the Emotional Empathic Drive Short Scale (EED), a five-item instrument grounded in Zaki’s (2014) theoretical framework of motivated empathy (Karlstetter, 2017). The EED measures individuals’ dispositional drive to share others’ emotions, capturing affective components of empathy rather than cognitive ones. Unlike traditional agree–disagree scales, this scale employs a frequency-based response format adapted from Spreng et al. (2009), which asks participants how often they feel or behave in ways described by each item. Each item is scored on a scale from 0 to 4, producing a total sum score ranging from 0 to 20, with higher scores indicating stronger emotional empathic drive.

This response format helps mitigate social desirability bias by focusing on the regularity of empathic experiences rather than levels of agreement, a concern raised in previous survey research (Karlstetter, 2015). It also helps preserve the multidimensionality of empathy more effectively than standard Likert scales. The EED has been validated in cross-cultural samples from Canada and Germany and is particularly suited for use in psychology, neuroscience, and social science contexts. In this study, the scale was administered in its Chinese version to undergraduate students aged 18 and older. The Cronbach’s α of this scale is 0.89.

#### 2.3.3 PCL-5

In this study, we employed the PTSD Checklist for DSM-5 (PCL-5) to assess PTSD symptoms among Chinese university students following exposure to traumatic literature. The PCL-5 is a 20-item self-report measure designed to evaluate the presence and severity of PTSD symptoms as defined by the *Diagnostic and Statistical Manual of Mental Disorders*, *Fifth Edition* (DSM-5; American Psychiatric Association, 2013). Developed by the National Center for PTSD, the PCL-5 represents a significant revision of the original PCL, which was based on DSM-IV criteria and has been one of the most widely used PTSD assessment tools in clinical and research settings since its introduction in the 1990s (Weathers et al., 1993; Blevins et al., 2015; Ruggiero et al., 2003).

The PCL-5 incorporates major changes to the PTSD diagnostic criteria outlined in the DSM-5, including the addition of new symptoms and a reorganization of symptom clusters. The measure now assesses symptoms across four distinct clusters: intrusion symptoms (Criterion B, items 1–5), avoidance symptoms (Criterion C, items 6–7), negative alterations in cognition and mood (Criterion D, items 8–14), and alterations in arousal and reactivity (Criterion E, items 15–20). Each item corresponds to a specific symptom, and respondents rate the degree to which they have been bothered by that symptom over the past month using a 5-point Likert scale ranging from 0 (“Not at all”) to 4 (“Extremely”).

Psychometric evaluations have demonstrated that the PCL-5 exhibits strong internal consistency (Cronbach’s α > 0.90), good test–retest reliability (*r* > 0.80), and excellent convergent and discriminant validity compared to other established PTSD measures (Blevins et al., 2015; Bovin et al., 2016). Additionally, the PCL-5 shows high sensitivity to clinical change, making it a valuable tool for monitoring treatment outcomes (Wortmann et al., 2016). In this study, the Cronbach’s α of the scale is 0.89.

### 2.4 Data analysis

The mediation model was tested based on cross-sectional data collected at the post-intervention stage (T1). Statistical analyses were performed using SPSS. First, descriptive statistics were calculated for all variables, including means, standard deviations, and frequencies for demographic characteristics across the three intervention groups. To assess baseline equivalence among groups, one-way analysis of variance (ANOVA) was used for continuous variables and chi-square tests for categorical variables.

The primary analysis examined changes in PTSD symptoms following the reading interventions. Paired sample t-tests were conducted to evaluate within-group differences in PCL-5 scores between T0 and T1 measurements for each of the three groups. To compare the relative effectiveness of the interventions, a one-way ANOVA was performed on the T1 scores, with post-hoc analyses to identify specific between-group differences.

To investigate the psychological mechanisms underlying the intervention effects, path analysis was employed to test the hypothesized mediation model. The model fit was evaluated using multiple goodness-of-fit indices. For the mediation analysis, we examined direct and indirect effects. Path coefficients were estimated for: (a) the effect of reading self-efficacy on empathy, (b) the effect of empathy on PTSD symptoms, (c) the total effect of reading self-efficacy on PTSD symptoms, and (c’) the direct effect of reading self-efficacy on PTSD symptoms controlling for empathy. The significance of the indirect effect was assessed to determine whether empathy mediated the relationship between reading self-efficacy and PTSD symptom reduction. Statistical significance was set at *p* < 0.05 for all analyses.

## 3 Results

### 3.1 Descriptive statistics

Table 2 presents the baseline demographic and psychological characteristics of participants across the three intervention groups. The mean age was similar across groups, indicating comparable age distribution. Gender composition was also balanced, with female participants accounting for 54.29% in Group 1, 51.43% in Group 2, and 60.00% in Group 3. In terms of academic standing, all groups included students across the four undergraduate years.

**Table 2:** Descriptive statistics.

Variable	Mean/%	Group 1	Group 2	Group 3
Age	Mean	20.03	19.71	20.23
Gender	Count (%)			
Female		19 (54.29)	18 (51.43)	21 (60.0)
Male		16 (45.71)	17 (48.57)	14 (40.0)
Grade	Count (%)			
Freshman		8 (22.86)	6 (17.14)	11 (31.43)
Sophomore		10 (28.57)	11 (31.43)	10 (28.57)
Junior		10 (28.57)	8 (22.86)	4 (11.43)
Senior		7 (20)	10 (28.57)	10 (28.57)
Reading self-efficacy	Mean ± SD	3.62 ± 0.50	3.76 ± 0.25	2.79 ± 0.24
Empathy	Mean ± SD	3.54 ± 0.76	3.49 ± 0.34	3.23 ± 0.16
PTSD	Total score	31–33		

Regarding baseline psychological measures, participants in Group 1 and Group 2 reported higher levels of reading self-efficacy (*M* = 3.62, SD = 0.50 and *M* = 3.76, SD = 0.25, respectively) compared to Group 3 (*M* = 2.79, SD = 0.24). Similarly, mean empathy scores were slightly higher in the intervention groups (Group 1: *M* = 3.54, SD = 0.76; Group 2: *M* = 3.49, SD = 0.34) than in the control group (*M* = 3.23, SD = 0.16). All participants scored within the 31–33 range on the PCL-5, confirming that they met inclusion criteria for exhibiting mild to moderate PTSD symptoms at baseline. These descriptive statistics suggest that the three groups were generally comparable at the start of the study in terms of demographics and key psychological variables.

### 3.2 Intervention effects on PTSD

**Figure 1:**
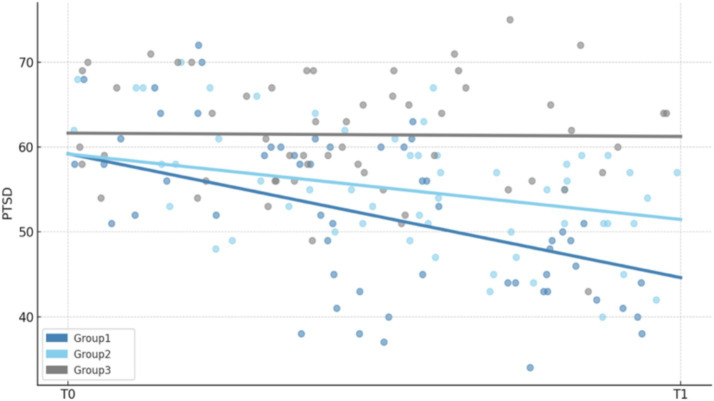
Changes in PTSD total scores (T0 to T1) across the three intervention groups.

To assess the effect of different reading interventions on reducing undergraduates’ PTSD symptoms, the current study compared PTSD scores across three groups at T0 and T1. A one-way ANOVA was conducted to compare the effects of the three intervention groups on T1 scores. The results (Table 3) revealed a statistically significant difference among the groups, *F*(2, 102) = 73.01, *p* = 0.02. Group 1 exhibited the lowest mean T1 score (*M* = 20.54, SD = 5.20), followed by Group 2 (*M* = 26.57, SD = 1.99) and Group 3 (*M* = 30.89, SD = 2.23). Paired-sample *t*-tests comparing pre-test (T0) and post-test (T1) scores showed significant improvements across all groups. Group 1 demonstrated the greatest change from T0 to T1 (*M* = −11.20, SD = 5.20), *t*(34) = 12.74, *p* < 0.001. Group 2 showed a moderate improvement (*M* = −5.34, SD = 2.27), *t*(34) = 13.90, *p* < 0.001, while Group 3 exhibited a smaller but still significant change (*M* = −1.03, SD = 2.29), *t*(34) = 2.65, *p* = 0.01. Figure 1 displays the distribution of total PTSD scores for the three groups across the two measurement points (T0 and T1), showing the most pronounced reduction in the trauma literature group.

**Table 3:** Pre- and post-intervention PTSD scores across groups and comparison results.

Group	T1 ± SD	Compare among groups	T1–T0	T0 vs T1 (*t*, *p*)	Cohen’s d
Group 1	20.54 ± 5.20	*F*(2, 102) = 73.01, *p* = 0.02*	−11.20 ± 5.20	*t* = 12.74 *p* = 0.00***	2.15
Group 2	26.57 ± 1.99		−5.34 ± 2.27	*t* = 13.90 *p* = 0.00***	2.35
Group 3	30.89 ± 2.23		−1.03 ± 2.29	*t* = 2.65 *p* = 0.01*	0.45

*p < 0.05; **p < 0.01; ***p < 0.001.

One-way ANOVA and chi-square tests were conducted to examine potential baseline differences among the three groups. The results showed no significant differences in age, gender, grade, or baseline PTSD symptoms (all *p* > 0.05), indicating that randomization successfully produced comparable groups before the intervention. Reading self-efficacy and empathy also showed no meaningful baseline differences across groups.

Figure 1 shows the distribution of total PTSD scores of the three groups of subjects at T0 and T1. The overall score of Group 1 decreased significantly, Group 2 decreased to a certain extent, and Group 3 had almost no change, indicating that there were significant differences in the intervention effects among the three groups.

### 3.3 Path analysis

We evaluated the goodness of fit of the research framework. According to Table 4, the research framework was deemed suitable for the collected data.

**Table 4:** Goodness of fit test.

Fit index	Acceptable values	Value
Chi-square		84.26
d.f.		62.0
Chi-square/d.f.	1–5	1.98
SRMR	0.08 or below	0.063
RMSEA	0.05–0.08	0.058
TLI	0.9 or above	0.932
CFI	0.9 or above	0.946

**Figure 2:**
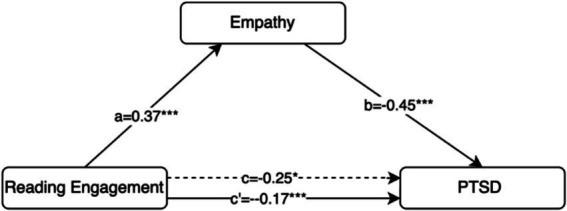
Mediation model illustrating the indirect effect of empathy between reading self-efficacy and PTSD symptoms.

A mediation analysis was conducted to examine whether empathy mediates the relationship between reading self-efficacy and PTSD symptoms (Figure 2). According to Table 5, reading self-efficacy was significantly positively associated with empathy, β = 0.37, *p* < 0.001, suggesting that individuals with greater reading self-efficacy tend to report higher levels of empathy. In turn, empathy was significantly negatively associated with PTSD symptoms, β = −0.45, *p* < 0.001, indicating that greater empathy is related to fewer PTSD symptoms. The total effect of reading self-efficacy on PTSD symptoms was significant, β = −0.25, *p* = 0.02. When empathy was included as a mediator, the direct effect of RS on PTSD symptoms (path c’) was reduced yet remained significant, β = −0.17, *p* < 0.001. These results suggest a partial mediation effect, supporting the hypothesis that empathy serves as an intermediary mechanism through which reading self-efficacy influences PTSD symptoms.

**Table 5:** Path coefficients for the mediation model.

Path	β	*p*
Path a (RS → EM)	0.37	0.00***
Path b (EM → PTSD)	−0.45	0.00***
Path c (RS → PTSD)	−0.25	0.02*
Path c’ (RS → PTSD)	−0.17	0.00***

## 4 Discussion

The results of this randomized controlled trial demonstrate that reading interventions can significantly impact PTSD symptoms among university students, with several important findings meriting discussion. First, our results clearly indicate that trauma-focused literature (Group 1) produced the most substantial reduction in PTSD symptoms compared to non-trauma literature (Group 2) and no intervention (Group 3). The significant difference between Group 1 and other groups suggests that engaging with narratives related to trauma may provide therapeutic benefits for individuals experiencing PTSD symptoms (O'Kearney and Perrott, 2006).

The stronger effect observed in Group 1 compared with Group 2 may reflect different psychological mechanisms. Trauma narratives such as *The Diary of Anne Frank* provide controlled emotional exposure, allowing readers to confront distressing themes and reappraise traumatic experiences in a safe context. This process resembles exposure-based therapy and may lead to deeper emotional processing and symptom reduction. This aligns with exposure-based theories in trauma treatment, where engagement with trauma-related content can facilitate processing and integration of traumatic experiences (McLean et al., 2022; Becker and Zayfert, 2001; Singewald et al., 2015). The marked improvement in Group 1 represents a clinically significant change, suggesting that guided reading of trauma narratives offers more than just statistical improvement but meaningful symptom relief.

Second, even non-trauma literature produced moderate but significant improvements in PTSD symptoms compared to no intervention. Group 2 demonstrated that engagement with literary narratives, even those not directly addressing trauma, can provide therapeutic benefits. This supports the therapeutic value of reading as an intervention, potentially operating through mechanisms such as emotional regulation (Arslan et al., 2022b; Milaré et al., 2021; Michalek et al., 2021) or perspective-taking (Bientzle et al., 2024) that may benefit mental health regardless of content specificity. The difference between Group 2 and Group 3 highlights that structured reading programs offer advantages beyond the natural recovery that might occur over time.

The significant reduction in PTSD symptoms across intervention groups aligns with our path analysis findings, suggesting that the reading interventions work through enhancing reading self-efficacy, which increases empathy and subsequently reduces PTSD. Reading self-efficacy appears to influence PTSD symptom reduction partially through increased empathy. This suggests that the confidence in one’s reading abilities may enhance engagement with the text, facilitating greater empathic responses to characters and situations (McCreary and Marchant, 2017), which in turn supports emotional processing related to trauma. The partial mediation effect indicates that while empathy plays a crucial role, reading self-efficacy also directly impacts PTSD symptoms through additional mechanisms. The strength of these relationships in our model suggests that fostering reading self-efficacy could be a viable approach to enhancing the effectiveness of bibliotherapy interventions.

## 5 Conclusion

Through a randomized controlled trial comparing trauma-focused literature, non-trauma literature, and no intervention conditions, we demonstrated that structured reading programs can significantly improve psychological wellbeing in individuals experiencing mild to moderate PTSD symptoms.

Trauma-focused literature produced the most substantial symptom reduction, supporting the value of exposure-based approaches in trauma treatment. However, non-trauma literature also yielded significant benefits compared to no intervention, suggesting that general reading interventions may offer therapeutic value through multiple psychological mechanisms. Additionally, the differential effects between trauma-focused and non-trauma literature indicate that tailoring reading materials to individual needs and preferences may optimize outcomes.

The mediation analysis further illuminated that reading self-efficacy influences PTSD symptom reduction partially through enhanced empathy, highlighting important psychological processes underlying bibliotherapy’s effectiveness. This mechanistic understanding can inform the development of more targeted reading interventions for trauma.

## 6 Limitations and future implications

Several limitations should be acknowledged. The relatively small sample size and specific demographic limit generalizability to broader populations with PTSD (Van De Schoot et al., 2015). Second, the 8-week timeframe may not capture longer-term effects or potential delayed while we controlled for baseline PTSD severity, other factors such as prior trauma history, reading habits, or personality traits might influence treatment response and were not fully accounted for in our analysis.

The findings suggest that guided reading programs can be integrated into existing psychological support systems for college students as a low-intensity intervention. Clinicians or counselors could recommend trauma-related literature as a structured exposure tool for clients with mild PTSD symptoms, accompanied by reflective writing or brief discussion sessions to facilitate emotional processing. Non-trauma literature can be used for stress reduction and emotional regulation among students with subclinical distress. Furthermore, reading self-efficacy could be incorporated as an assessment indicator to tailor intervention intensity and monitor progress. These practices can help bridge the gap between traditional therapy and accessible, literature-based mental health support in university or community settings.

Future research should explore the durability of these effects through longer follow-up periods, investigate whether benefits extend to individuals with more severe PTSD presentations, and examine if specific trauma types respond differently to various reading materials. Additionally, future studies could integrate reflective writing, guided group sessions, or narrative sharing to enhance emotional processing and empathy development.

## Data Availability

The raw data supporting the conclusions of this article will be made available by the authors, without undue reservation.
